# Unexpected fibrous mediastinitis in a patient with myasthenia gravis - a case report

**DOI:** 10.1186/s13019-023-02417-9

**Published:** 2023-11-14

**Authors:** Xuyang Wang, Xiaoping Zuo, Fuqiang Wang, Yun Wang

**Affiliations:** 1 Department of Thoracic Surgery, West China Hospital, Sichuan University, No. 37, Guoxue Alley, Chengdu, Sichuan 610041 China; 2 Department of Thoracic Surgery, Guang’an People’s Hospital, Guangan, Sichuan 638000 China

**Keywords:** Fibrous mediastinitis, Myasthenia gravis, Autoimmune disease

## Abstract

**Background:**

Fibrous mediastinitis (FM) is a rare mediastinal lesion characterized by proliferation of fibrous tissue within the mediastinum. Previous reports have shown that this lesion can be caused by histoplasmosis and tuberculosis. In extremely rare cases, FM can also be caused by autoimmune diseases such as antineutrophil cytoplasmic antibody-associated vasculitis and large-vessel arteritis.

**Case presentation:**

In our case, we report unexpected fibrous mediastinitis found after robotic thymectomy in a patient with myasthenia gravis (MG). The preoperative imaging indicated no obvious lesion in the mediastinum and the patient denied histories of both histoplasmosis and tuberculosis. After the operation, both proliferation of fibrous tissue and ectopic germinal centres (GCs) could be found in the thymus.

**Conclusion:**

This rare case might enrich our knowledge of the relationship between FM and autoimmune diseases.

## Background

Fibrous mediastinitis (FM) is a rare mediastinal lesion characterized by proliferation of fibrous tissue within the mediastinum. It is generally thought that FM was an abnormal host response related to previous infection with histoplasma and tuberculosis [1]. In rare cases, however, some FM patients have no history of infection and are accompanied by several autoimmune disease markers and fibroinflammatory lesions. Thus, FM is classified as having either secondary (secondary to infection) or idiopathic (without clear cause) forms [2].

Myasthenia gravis (MG) is one kind of autoimmune disease that affects the neuromuscular junction. The antibody (Ab) targeted to the acetylcholine receptor (AChR) is the main cause of MG. Previous research has shown that MG is closely related to thymic lesions including thymoma and thymic hyperplasia [3, 4]. However, we found no reports of FM in a MG patient.

Herein, we present a rare case of unexpected FM found in a patient with MG who had undergone robotic thymectomy. This case might enhance our knowledge of the relationship between FM and autoimmune diseases.

## Case presentation

A female aged 56 years was admitted for treatment of generalized MG. Her chief complaints were ptosis, dysphagia, and upper limb weakness for three years. Initially, the manifestation of MG started with ocular symptoms, including left-eyelid ptosis and double vision. Four months later, she presented with left upper limb weakness, swallowing disorders, and dysphonia. Results of enzyme-linked immunosorbent assay (ELISA) showed that the AChR-Ab was positive (38.79 nmol/L) and the muscle-specific kinase antibody (MuSK-Ab) was negative (0.03 U/mL). She denied a history of tuberculosis or other bacterial infections. Based on her clinical symptoms and laboratory test, she was diagnosed with type IIb MG [5] and treated with oral administration of pyridostigmine (60 mg three times daily), prednisone (15 mg once daily), and tacrolimus (1 mg once daily). After treatment, the ocular symptoms were largely improved but the swallowing disorders and limb weakness continued to affect her quality of life to a significant extent. She was therefore admitted for thymectomy.

**Fig. 1:**
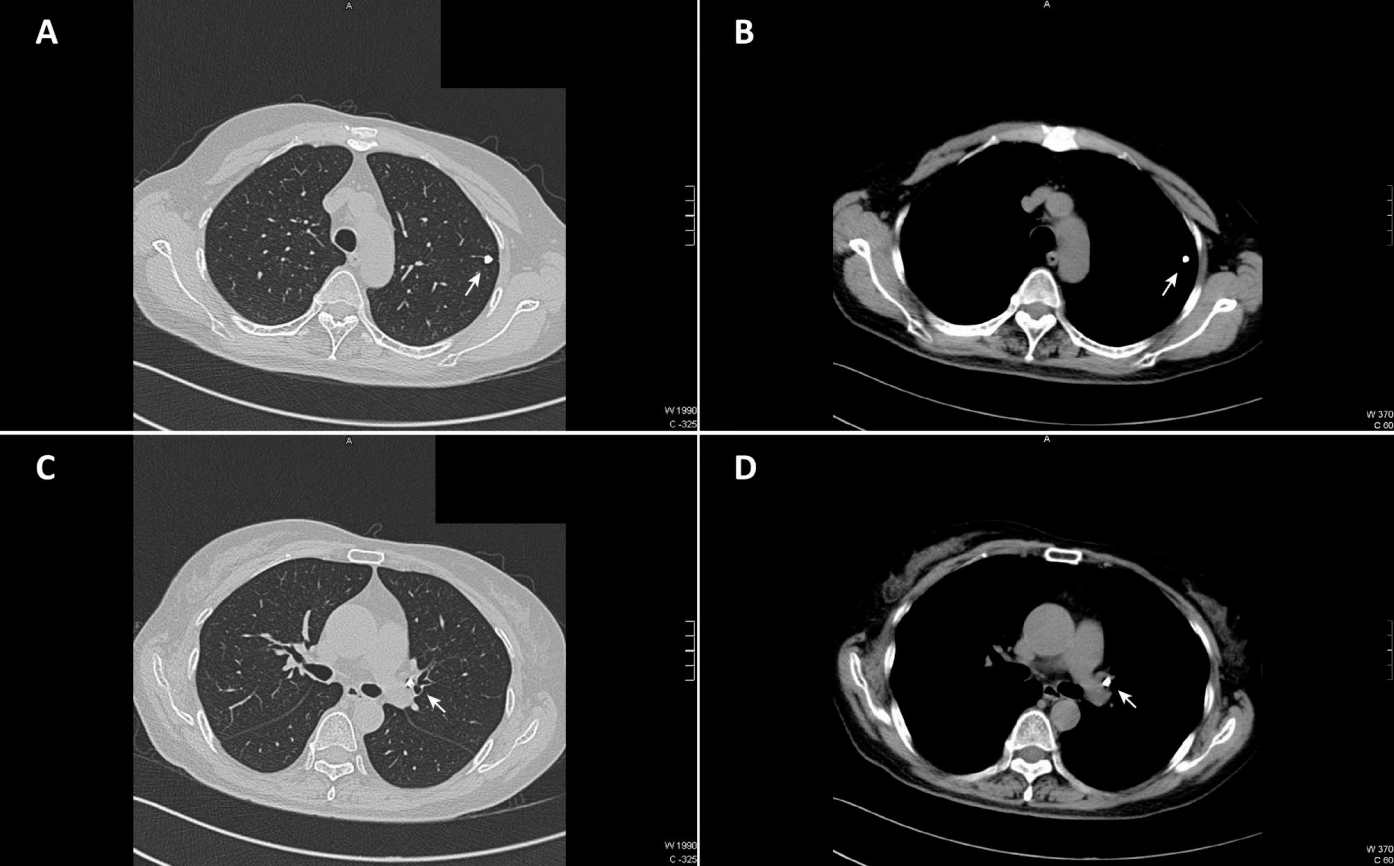
Chest CT findings. There were no overt imaging abnormalities. Only sporadic calcifications were found in the left lung (**A**, **B**; white arrows) and near left hilar regions (**C**, **D**; white arrows)

After this admission, no ocular symptoms were noted upon physical examination. The Myasthenia Gravis Composite score was 4 (2 for swallowing and 2 for shoulder abduction). Routine blood test, urinalysis, and liver function, renal function, and coagulation tests were all within normal ranges. In chest CT, only sporadic calcifications were detected in the lymph node near the left hilum and in the left upper lung (Fig. 1).

**Fig. 2:**
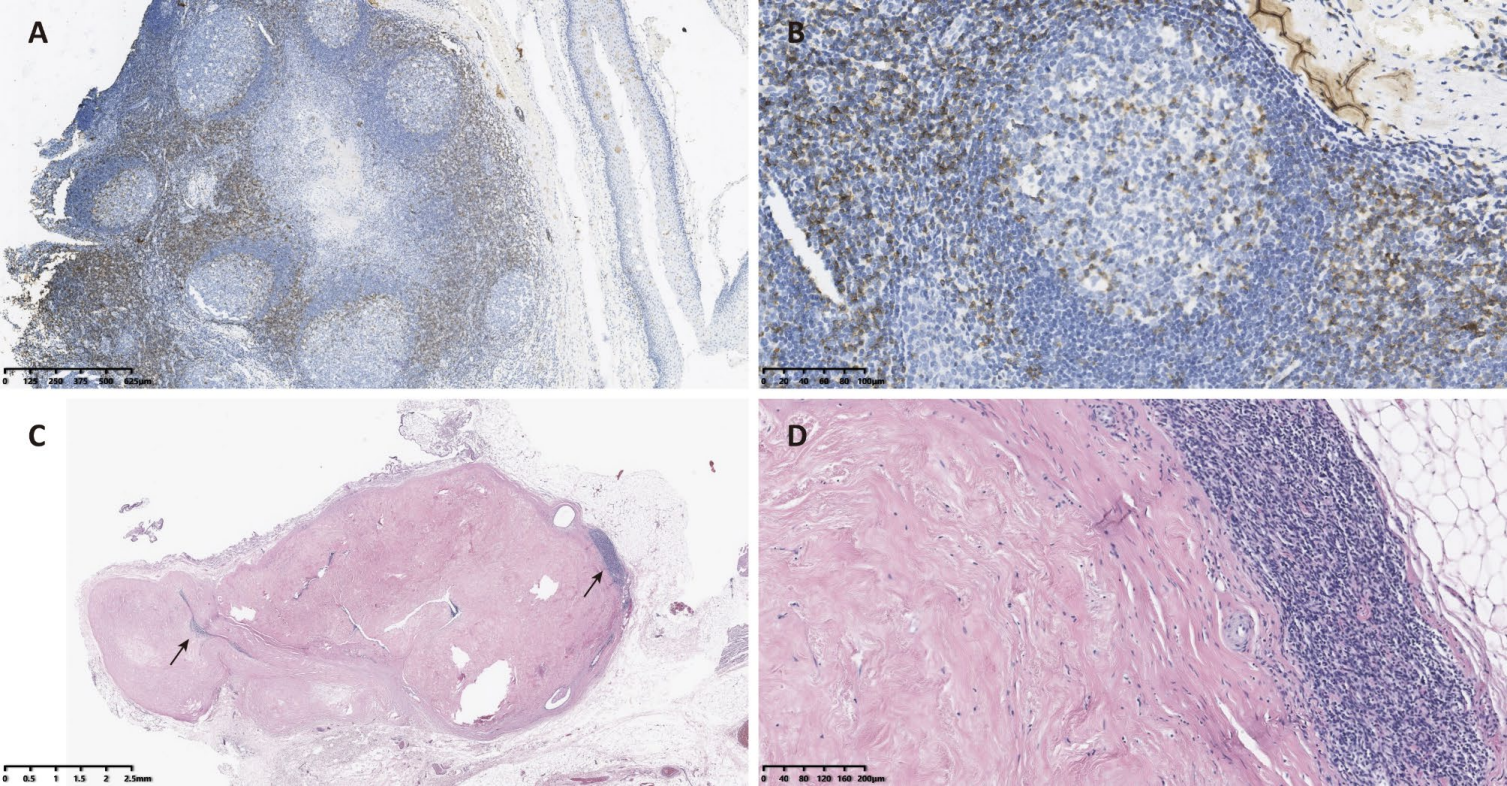
Thymic pathology findings. (**A** and **B**), IHC staining for CD5 (anti-CD5 antibody, ZA-0510, ZSGB-BIO, China) shows that several ectopic GCs can be found in the thymic cortex (A, ×4; B, ×20). (**C** and **D**), HE-stained images show fibrous tissue proliferation and infiltrated lymphocytes (C, ×1; D, ×10). The lymphocytes were mainly located in the peripheral region of fibrosing proliferation (black arrows)

Rrobotic transxiphoid thymectomy was performed to remove the thymus and perithymic adipose tissue [6]. Interestingly, in addition to the signs of MG, a pathological feature of FM was also found on final pathology. As shown in Fig. 2, numerous ectopic GCs were identified in the thymic cortex by immunohistochemistry (IHC) [7]. In addition, dense collagen was distributed in a haphazard pattern and was infiltrated with minimal numbers of inflammatory cells; this matches features of stage II fibrosing mediastinitis [8, 9]. As a result, the final diagnosis of this patient was generalized MG (IIb) and FM.

After the operation, the patient recovered uneventfully and was discharged on postoperative day 3. The postoperative T-SPOT test for tuberculosis was negative. After follow-up for 16 months, the patient had achieved pharmacological remission of MG and oral pyridostigmine has been discontinued. The patient is now treated with prednisone (10 mg every other day) and tacrolimus (1 mg once daily). Written informed consents for therapy and for the case report were obtained from the patient.

## Disscussion and conclusion

In most cases, FM patients are more likely to present with symptoms associated with compression of mediastinal structures. The common manifestation of FM includes the head fullness and facial swelling caused by compression of the superior vena cava and dyspnoea caused by compression of airways and pulmonary veins [1]. In chest imaging, calcification can be found in 86% of patients. Mediastinal structures including the superior vena cava, central airways, and pulmonary veins can be compressed, which leads to vascular stenoses and pulmonary atelectasis, and the soft tissue mass compressing the mediastinal structure [10]. In our case, asymptomatic FM was found unexpectedly and there were no overt imaging abnormalities in chest CT. We surmised that the oral immunosuppressive agent used to treat the MG could suppress thymic inflammation, and thus repress the development of FM.

Flieder et al. and other have previously subdivided the idiopathic fibroinflammatory lesions of the mediastinum into three groups based on the histologic pattern of this type of lesion [8, 9]. According to this staging system, as the lesion stage increases, the dense collagen component also increases but the infiltration of inflammatory cells is reduced. In our case, dense collagen was distributed in a haphazard pattern and was infiltrated with minimal numbers of inflammatory cells. There was no obvious oedematous fibromyxoid tissue. Thus, the pathological pattern found in our case is consistent with the features of stage II fibrosing mediastinitis.

The pathogenesis of FM is largely obscure. However, previous research has shown that FM is associated with several autoimmune diseases [2]. In a case series of nine idiopathic FM (IFM) patients by Rossi et al. [2], seven patients were associated with autoimmune or fibro-inflammatory disorders including antineutrophil cytoplasmic antibody-associated vasculitis and aortitis. Further research has shown that several immune cells including monocytes and B cells, and in particular, CD20-plasma cells, were infiltrated in the peripheral region of fibrosing proliferation [1, 2, 11]. In our case, lymphocyte infiltration was also identified around the fibrosing tissue. Because of the thymic inflammation and overactive immune responses in development of FM, anti-inflammatory therapy such as that involving administration of oral prednisone was applied to treat the FM [1]. Recently, rituximab targeting CD20^+^ B cells was proven to be effective in treating FM [11]. In consideration of the close association and similar treatment between FM and autoimmune disease, Rossi et al. classified the “IFM associated with systemic autoimmune diseases” as one of the three forms of IFM [2]. In our case, while the patient had been diagnosed with MG, she denied a history of previous bacterial infection. However, due to the unexpectedness of this case, testing for tuberculosis and histoplasma was not undertaken before surgery. The postoperative T-SPOT test for tuberculosis was negative. Despite this shortcoming, this rare case of asymptomatic FM found in a patient with extended our knowledge on autoimmune disease-associated FM.

## Data Availability

Not applicable.

## List of abbreviations

FM: Fibrous mediastinitis
IFM: Idiopathic fibrous mediastinitis
MG: Myasthenia gravis
GC: Germinal centre
IHC: Immunohistochemistry
CT: Computed tomography
AChR: Acetylcholine receptor
MuSK: Muscle-specific kinase
Ab: Antibody
ELISA: Enzyme-linked immunosorbent assay

## References

[1] Peikert T, Colby TV, Midthun DE, Pairolero PC, Edell ES, Schroeder DR, Specks U (2011). Fibrosing mediastinitis: clinical presentation, therapeutic outcomes, and adaptive immune response. Med (Baltim).

[2] Rossi GM, Emmi G, Corradi D, Urban ML, Maritati F, Landini F, Galli P, Palmisano A, Vaglio A (2017). Idiopathic Mediastinal Fibrosis: a systemic Immune-mediated disorder. A Case Series and a review of the literature. Clin Rev Allergy Immunol.

[3] Gilhus NE, Verschuuren JJ (2015). Myasthenia gravis: subgroup classification and therapeutic strategies. Lancet Neurol.

[4] Marx A, Pfister F, Schalke B, Saruhan-Direskeneli G, Melms A, Ströbel P (2013). The different roles of the thymus in the pathogenesis of the various myasthenia gravis subtypes. Autoimmun Rev.

[5] Jaretzki A, Barohn RJ, Ernstoff RM, Kaminski HJ, Keesey JC, Penn AS, Sanders DB (2000). Myasthenia gravis: recommendations for clinical research standards. Task Force of the Medical Scientific Advisory Board of the Myasthenia Gravis Foundation of America. Neurology.

[6] Zhang H, Wang F, Qiu G, Li Z, Chen LQ, Wang Y (2022). Surgical Tips to improve completeness of Transsubxiphoid Robotic Extended Thymectomy. Ann Thorac Surg.

[7] Ramos-Vara JA. Principles and methods of immunohistochemistry. Drug Saf Evaluation: Methods Protocols 2011:83–96. 10.1007/978-1-60761-849-2_5. 10.1007/978-1-60761-849-2_5 20972748

[8] Flieder DB, Suster S, Moran CA (1999). Idiopathic fibroinflammatory (fibrosing/sclerosing) lesions of the mediastinum: a study of 30 cases with emphasis on morphologic heterogeneity. Mod Pathol.

[9] Lindholm KE, de Groot P, Moran CA (2019). Fibrosing/Sclerosing lesions of the Mediastinum: a review. Adv Anat Pathol.

[10] Rossi SE, McAdams HP, Rosado-de-Christenson ML, Franks TJ, Galvin JR (2001). Fibrosing mediastinitis. Radiographics.

[11] Westerly BD, Johnson GB, Maldonado F, Utz JP, Specks U, Peikert T (2014). Targeting B lymphocytes in Progressive fibrosing mediastinitis. Am J Respir Crit Care Med.

